# Efficacy of eyelid warming therapies in contact lens-related dry eye and comfortable wearing time: a systematic review with meta-analysis

**DOI:** 10.1007/s44402-026-00024-4

**Published:** 2026-02-27

**Authors:** Antonio Ballesteros-Sánchez, Carlos Rocha-de-Lossada, Davide Borroni, Rasaa Larhlid-Bouzouad, José-María Sánchez-González

**Affiliations:** 1 School of Optics and Optometry, Universitat Politècnica de Catalunya, Terrassa, Spain; 2 Ophthalmology Department, VITHAS Malaga, Malaga, Spain; 3 Regional University Hospital of Malaga, Hospital Civil Square, Malaga, Spain; 4 Qvision, Ophthalmology Department, VITHAS Almeria Hospital, Almeria, Spain; 5 Surgery Department, Ophthalmology Area, University of Seville, Seville, Spain; 6 Riga Stradins University, Riga, Latvia; 7 Centro Oculistico Borroni, Gallarate, Italy; 8 Eyemetagenomics Ltd., London, UK; 9 Department of Physics of Condensed Matter, Optics Area, Pharmacy Faculty, University of Seville, Seville, Spain; 10 Present Address: Department of Physics of Condensed Matter, Optics Area, Pharmacy Faculty, University of Seville, Seville, Spain

**Keywords:** contact lens-related dry eye, contact lens wearing time, eyelid warming therapies, lipiflow, warm compress

## Abstract

**Purpose:**

To analyse the efficacy and safety of eyelid warming therapies in the management of contact lens-related dry eye (CLDE) and their impact on comfortable contact lens (CL) wearing time.

**Methods:**

A systematic review with meta-analysis, reporting on the efficacy and/or safety of eyelid warming therapies for CLDE and/or comfortable CL wearing time in three databases, PubMed, Scopus and Web of Science, was performed according to the preferred reporting items for systematic reviews and meta-analyses (PRISMA) statement.

**Results:**

Four randomised controlled trials were included. Analyses revealed significant differences only between eyelid warming therapies and negative controls for ocular surface disease index scores (mean difference (MD): 19.57; 95% CI: 12.51–26.64; *I*^2^ = 0%; *p* < 0.001), with *LipiFlow*® and warm compresses showing comparable results (*p* = 0.53*)*. Warm compresses did not achieve significant improvements in tear film break-up time (MD: 0.80; 95% CI: −0.29 to 1.88; *I*^2^ = 0%; *p* = 0.15), lipid layer thickness (MD: 4.54; 95% CI: −5.73 to 14.81; *I*^2^ = 0%; *p* = 0.39) or comfortable CL wearing time (MD: −0.06; 95% CI: −1.19 to 1.06; *I*^2^ = 0%; *p* = 0.91) when compared with positive controls, nor in the Schirmer test when compared with negative/positive controls (MD: −0.46; 95% CI: −4.17 to 3.25; *I*^2^ = 24%; *p* = 0.81). Regarding safety, most studies reported no adverse events and demonstrated a satisfactory tolerability profile for eyelid warming therapies. However, a meta-analysis could not be performed.

**Conclusions:**

The efficacy of eyelid warming therapies for alleviating CLDE and improving comfortable CL wearing time is limited. Further randomised controlled trials following the consolidated standards of reporting trials (CONSORT) guidelines are needed to confirm these findings.

Key Points

- In contact lens wearers with meibomian gland dysfunction-related dry eye, modifications to contact lens parameters, wearing schedule or care systems may not adequately relieve contact lens-related dry eye or extend comfortable wearing time. In these cases, eyelid warming is considered a first-line therapy.
- Eyelid warming therapies appear to reduce ocular surface disease index scores compared to no intervention, with comparable results observed between LipiFlow® and warm compresses. However, the heterogeneity among the included studies, potential risk of bias, conflicts of interest and industry funding indicate that this result should be interpreted with caution.
- Based on the most current scientific literature, this systematic review with meta-analysis suggests that evidence remains insufficient to support the efficacy of eyelid warming therapies for alleviating contact lens-related dry eye and improving comfortable contact lens wearing time, as well as to establish their safety profile adequately.

## Introduction

Contact lenses (CL) are widely used for vision correction and cosmetic purposes [1, 2], offering significant improvements in the quality of life of CL wearers worldwide [3]. Despite these benefits, CL discomfort remains a persistent challenge in eye care practices, with studies consistently reporting its prevalence in approximately 50% of CL wearers [4].

In 2013, CL discomfort was defined as “*a condition characterised by episodic or persistent adverse ocular sensations related to CL use, either with or without visual disturbance, resulting from reduced compatibility between the CL and the ocular environment, which can lead to decreased wearing time and discontinuation*” by the Tear Film and Ocular Surface (TFOS) International Workshop on CL discomfort [5]. Subsequently, the CL Materials, Design and Care Subcommittee reviewed the evidence for a range of strategies for its management, concluding that refitting CL wearers with an alternative replacement schedule, care system, material and design—specifically considering edge design and lens movement—can enhance comfort [6]. However, CL wearers often present with signs and symptoms of dry eye associated with meibomian gland dysfunction (MGD) [7–9]. In such cases, modifications to the wear regimen, maintenance systems and CL parameters are insufficient to alleviate contact lens-related dry eye (CLDE) [10]. Therefore, it is essential to identify and treat any eyelid margin disease prior to CL fitting [11], as MGD may be exacerbated by CL use, further intensifying discomfort [11, 12].

Previous studies have demonstrated that treating MGD enhances tear film stability, leading to an increase in comfortable CL wear time [13, 14]. Since MGD was defined as “*a chronic, diffuse anomaly of the meibomian glands, often characterised by terminal duct blockage and/or qualitative/quantitative changes in meibum secretion*” by the TFOS International Workshop on MGD [15], treatments have aimed at removing obstructions from the terminal ducts, thereby improving meibomian gland function [16, 17]. Among these, eyelid warming remains a first-step therapy, as the application of heat increases liquefaction of meibum and promote its release from the gland orifice [17]. Several eyelid warming therapies are available, including warm compresses, iLux® (Alcon Inc., ilux.myalcon.com), LipiFlow® (TearScience Inc., lipiflow.jnjvisionpro.com) and TearCare® (Sight Sciences Inc., tearcare.sightsciences.com). Currently, literature reviews suggest that eyelid warming therapies may improve the signs and symptoms of MGD-related dry eye, with a satisfactory safety profile [18–21]. Furthermore, most randomised controlled trials (RCTs) have been conducted on non-CL wearers [22–25]. However, to the best of our knowledge, no studies have systematically reviewed the efficacy and safety of these therapies in CL wearers with MGD-related dry eye. Therefore, this study aims to analyse the efficacy and safety of eyelid warming therapies in CLDE and their effect on comfortable CL wearing time.

## Methods

### Data Sources and Search Strategy

This systematic review with meta-analysis (PROSPERO ID: CRD42025634720) was conducted in accordance with the Preferred Reporting Items for Systematic Reviews and Meta-Analyses (PRISMA) [26]. The search strategy was guided by the following PICO (i.e., patient/population/problem, intervention, comparison and outcome) questions: (1) *Are eyelid warming therapies effective and safe in patients with contact lens–related dry eye?* and (2) *Do eyelid warming therapies influence comfortable contact lens wearing time in patients with contact lens–related dry eye?*

To address this question, a comprehensive literature search was conducted across PubMed, Scopus and Web of Science using the following Boolean operators: (hot towel OR warm compress OR eyelid warming compress OR eyelid warming devices OR heated mask OR eyegiene mask OR MGDRx eyebag OR therapearl OR bruder moist heat compress OR meibopatch OR moisture chamber goggles OR blephasteam OR ilux OR vectored thermal pulsation OR VTP OR lipiflow OR tearcare) AND (contact lens OR contact lens wearers OR contact lens-associated dry eye OR contact lens discomfort OR contact lens wearing time). This search identified a total of 167 relevant articles published between April 2003 and June 2025.

### Study Selection

**Fig. 1:**
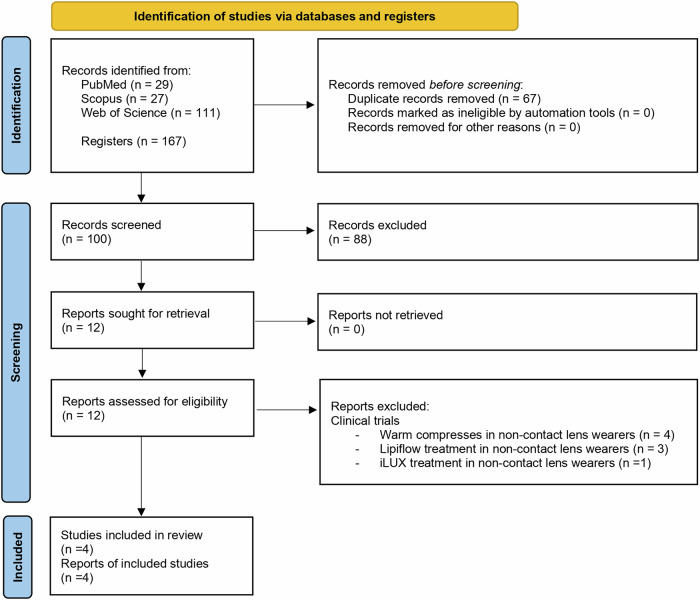
Flowchart of study selection process according to the PRISMA statement.

All of these 167 articles identified through the search strategy were considered and analysed. Duplicate studies were removed by Mendeley Reference Manager, version 2.128.0 (Elsevier Ltd., Mendeley.com) [27]. The remaining studies underwent additional screening stages, which included title, abstract and full-text screening. Studies unrelated to the topic were excluded from the review during title and abstract screening. The screening of the full-text studies was performed by two investigators (ABS and RLB), who selected them according to the inclusion and exclusion criteria. Inclusion criteria were as follows: full-length prospective RCTs conducted on humans, reporting on the efficacy and/or safety of eyelid warming therapies for CLDE and/or comfortable CL wearing time. Exclusion criteria included publications in languages other than English and/or non-indexed journals. There were no restrictions placed on the country in which the study was performed, the follow-up period, the sample size or results of the studies. A more detailed description of the study selection process is presented in Fig. 1.

### Data Extraction and Quality Assessment

The data from each study were collected and summarised independently in tables designed by two investigators (ABS and RLB). The following information was obtained from each article: (1) *Author and date of publication (year)*, (2) *Study design*, (3) *Mean follow-up of all patients in the whole procedure (expressed in months)*, (4*) Number of patients*, (5) *Mean age of the patients (expressed in years)*, (6) *Patient sex (male/female)*, (7) *Inclusion criteria of the studies*, (8) *Study group intervention*, (9) *Control group intervention and* (10) *Conflicts of interest*. Regarding the results of the studies, the following clinical parameters—identified as the most frequently reported across studies—were collected: (11) *Ocular Surface Disease Index (OSDI) questionnaire*, (12*) Tear film break-up time (TBUT) assessed with fluorescein (FTBUT) or through non-invasive methods (NIBUT)*, (13) *Schirmer test without anaesthesia*, (14) *Lipid layer thickness (LLT)* and (15) *comfortable CL wear time*.

The studies that remained after the full-text screening were evaluated further to determine their quality. Two investigators (CRDL and JMSG) evaluated the risk of bias independently using the Cochrane Risk of Bias 2 (RoB 2) tool (The Cochrane Collaboration, RoB2.cochrane.org) [28]. This tool evaluates the methodological quality of RCTs across five domains: (1) *Bias arising from the randomisatio7n process*, (2) *Bias due to deviations from intended intervention*, (3*) Bias due to missing outcome data*, (4) *Bias in measurement of the outcome* and (5) *Bias in selection of the reported result*. Each domain includes one or more signalling questions intended to guide the risk of bias assessment. Responses to these questions are categorised as *“yes,” “probably yes,” “probably no,” “no”* and *“no information.”* Based on predefined algorithms, RoB 2 translates these responses into a risk of bias judgement for each domain, classified as *“low risk of bias”*, *“some concerns”* or *“high risk of bias”*. The overall risk of bias for each RCT was determined by the highest level of risk assigned to any of the domains. However, if multiple domains were categorised as *“some concerns”*, then the study could also be judged as having a high overall risk of bias. A third non-masked investigator (DB) decided the quality of the studies when disagreements occurred between the two primary investigators (CRDL and JMSG).

### Data Synthesis and Analyses

Regarding data synthesis, both intra-group and inter-group outcome measures were reported, indicating whether the differences observed were statistically significant according to the analysis performed by the authors of each study. Intra-group outcome measures were presented as *“Last visit (LV) – Baseline (B) differences,”* while inter-group outcome measures were reported as *“Eyelid warming therapies*_*(Last visit – Baseline)*_ *– Control*_*(Last visit – Baseline)*_*.”*

Meta-analyses were conducted to pool inter-group outcome measures using the Review Manager (RevMan) Web, version 5.7 (The Cochrane Collaboration, RevMan.cochrane.org) [29]. Initially, the aim was to evaluate the overall efficacy and safety of eyelid warming therapies, followed by separate analyses of specific interventions—including warm compresses, LipiFlow®, iLux® and TearCare®—for CLDE and comfortable CL wearing time. However, due to the limited safety data reported in the studies included, combined with the small number of RCTs available for eyelid warming therapies in CL wearers, the meta-analyses were restricted to the following comparisons:

- Efficacy of eyelid warming therapies versus controls (negative/positive controls).
- Efficacy of warm compresses versus controls (negative/positive controls).

In addition, the following subgroup analysis was conducted:

- Efficacy of LipiFlow® versus warm compresses.

During these analyses, if there were different data measurement methods among the studies, then standardised mean differences (SMD) were calculated. SMD is a measure of the size of the intervention effect in each study with respect to the variability within the study, which allows analysis of the results on a uniform scale [30]. On the other hand, if there were no different data measurement methods among the studies, then the mean differences (MD) were calculated [30]. These effect measures were interpreted in conjunction with the *p*-value and the 95% confidence intervals (CI) presented on Forest plots. A *p* < 0.05 was considered statistically significant. Heterogeneity of the included studies was analysed together with the Cochrane Q-statistics chi-square test (*χ*^2^) and I-square test (*I*^2^) [31]. If there was any significant heterogeneity between studies (*χ*^2^ with a *p* < 0.05 or *I*^2^ > 50%) then a random effects model was performed to pool the data, otherwise a fixed effects model was conducted [32, 33]. Sensitivity analyses were also performed to evaluate the robustness and stability of the obtained results [32]. Given the use of both negative and positive control groups across the included studies, the following sensitivity analyses were performed:

- Efficacy of Eyelid Warming Therapies Versus Negative Controls.
- Efficacy of warm compresses versus positive controls.
- Efficacy of warm compresses versus first-step therapies recommended by TFOS DEWS II.

**Fig. 2:**
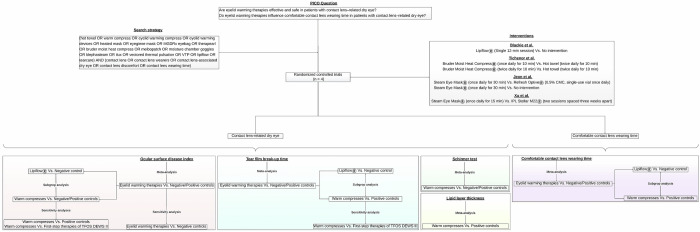
Flowchart of meta-analyses, subgroup analysis and sensitivity analyses. PICO, patient/population/problem, intervention, comparison and outcome questions; TFOS DEWS, Tear Film and Ocular Surface Dry Eye Workshop. Works cited are Blackie et al. [13], Tichenor et al. [14], Jeon and Park [34], and Xu et al. [35].

A negative control is a group that received no treatment and was not expected to produce any effect, whereas a positive control received a treatment that produced a known response. A flowchart detailing the analyses conducted for CLDE and comfortable CL wearing time is shown in Fig. 2.

## Results

### Study characteristics

This systematic review included four RCTs [13, 14, 34, 35] published between 2018 and 2022, comprising 304 eyes from 244 patients with a mean age of 26.6 ± 10.8 years. The sex distribution was 199 females (81.5%) and 45 males (18.5%). Patient follow-up, expressed in months, ranged from 1 [14, 34] to 3 months [13], with a mean follow-up of 1.4 ± 0.7 months. Regarding the study group interventions, one study performed LipiFlow® [13], while the remaining studies applied warm compresses—defined as eyelid masks that provide controlled and sustained heat to the eyelids—including Bruder Moist Heat Compress® (Bruder Healthcare Company, bruder-mask.bruder.com) [14] or the Steam Eye Mask® (Ocuface Medical Corporation Ltd., steameyemask.com) [34, 35]. The control groups included both negative and positive comparators, such as non-intervention, hot towels [14]—defined as towels or washcloths manually heated under hot water and applied to the eyelids, reflecting a variable and less controlled temperature—,artificial tears (Refresh Optive®, AbbVie Inc., refresheyedrops.com) [34] or intense pulse light (IPL) (Stellar M22®, Lumenis Be Ltd., lumenis.com).

Conflicts of interest were reported in two studies, attributed to affiliations with the manufacturers responsible for distributing the product under investigation [13, 14]. A more detailed description of the study characteristics is provided in Table 1.

**Table 1:** Summary of included RCTs comparing eyelid warming therapies Vs. Negative/Positive controls.

Author (date)	Design	FU^a^	Patients	Age^b^	Sex (M/F)	Eyes	Inclusion criteria	Eyelid warming therapies	Controls	CoI
Blackie et al. [13]. 2018	MT UM	3	55	41.7 ± 14.5	8/47	55	MGD diagnosis Positive CLDE symptoms Contact lens characteristics: • Soft daily CL • CLW 4 times/week for 2 hours/day	Lipiflow^®^ (single 12-min session)	No intervention	Yes
Tichenor et al. [14]. (A) 2019	MN UM	1	34	34 ± 11.5	0/34	34	Positive CLDE symptoms Contact lens characteristics: • Soft daily or reusable CL • CLW 4 times/week for 4 hours/day	Bruder Moist Heat Compress^®^ (once daily for 10 min)	Hot towel (twice daily for 10 min)	Yes
Tichenor et al. [14]. (B) 2019	MN UM	1	34	34 ± 9.5	1/33	34	Positive CLDE symptoms Contact lens characteristics: • Soft daily or reusable CL • CLW 4 times/week for 4 hours/day	Bruder Moist Heat Compress^®^ (twice daily for 10 min)	Hot towel (twice daily for 10 min)	Yes
Jeon and Park [34]. (A) 2020	MN SM	1	52	21.2 ± 2.1	6/46	52	Positive CLDE symptoms Contact lens characteristics: • Soft CL • CLW for 1 year	Steam Eye Mask^®^ (once daily for 30 min)	Refresh Optive^®^ (0.5% CMC, single-use vial once daily)	No
Jeon and Park [34]. (B) 2020	MN SM	1	52	21.5 ± 1.8	4/48	52	Positive CLDE symptoms Contact lens characteristics: • Soft CL • CLW for 1 year	Steam Eye Mask^®^ (once daily for 30 min)	No intervention	No
Xu et al. [35]. 2022	MN SM	1.5	60	28.6 ± 4.2	28/32	120	MGD diagnosis Contact lens characteristics: • Soft daily or reusable CL • CLW for 1 year	Steam Eye Mask^®^ (once daily for 15 min)	IPL Stellar M22^®^ (two sessions spaced three weeks apart)	No

*CL* contact lens, *CMC* carboxymethyl cellulose, *CLDE* contact lens-related dry eye, *CLW* contact lens wear, *CoI* conflict of interest, *SM* single-masked, *F* female, *FU* follow-up, *M* male, *MN* monocenter, *MT* multicenter, *RCTs* randomised controlled trials, *UM* unmasked.

^a^Expressed as months.

^b^Expressed as mean ± SD (standard deviation).

### Efficacy of Eyelid Warming Therapies

#### Contact lens-related dry eye

Intra-group and inter-group efficacy on CLDE are shown in Table 2. Overall, the studies reported significant improvements in most outcome measures after eyelid warming therapies.

**Table 2:** Intra-group and inter-group differences in CLDE of Eyelid warming therapies Vs. Controls.

	Eyelid warming therapies				Control				Inter-group differences^b^			
	OSDI, points	TBUT, s^i^	ST, mm	LLT, nm	OSDI, points	TBUT, s	ST, mm	LLT, nm	OSDI, points	TBUT, s	ST, mm	LLT, nm
Baseline	39.6 ± 16.4	4.8 ± 2.7	NR	NR	40.8 ± 20.3	4.6 ± 2	NR	NR				
Last visit	13.4 ± 15.5	6.5 ± 4	NR	NR	37.5 ± 23.8	4.3 ± 1.7	NR	NR				
**Difference**^**a**^	**−26.2** ± **20.3**	**1.7** ± **3.4**	**-**	**-**	**−3.3** ± **22.6**	**−0.3** ± **1.9**	**-**	**-**	−22.9^*^	2^*^	**-**	**-**
Baseline	33.8 ± 17.3	6.8 ± 2.8	NR	57.9 ± 21.4	27.1 ± 18.4	7.6 ± 4.2	NR	60.3 ± 23.9				
Last visit	17.5 ± 18.1	7 ± 2.7	NR	59.3 ± 22	18.6 ±	8.5 ± 3.7	NR	57 ± 22.6				
**Difference**^**a**^	**−16.3** ± 17.7^*^	**−0.2** ± **2.7**	**-**	**1.4** ± **21.7**	**−8.5** ± 14.5^*^	**−0.9** ± **0.6**	**-**	**−3.3** ± **23**	**−7.8**	**0.7**	**-**	**4.7**
Baseline	22.9 ± 11.9	7.4 ± 3.8	NR	57.1 ± 19.2	27.1 ± 18.4	7.6 ± 4.2	NR	60.3 ± 23.9				
Last visit	14 ± 14.7	7.3 ± 4	NR	58.2 ± 21.4	18.6 ±	8.5 ± 3.7	NR	57 ± 23.5				
**Difference**^**a**^	**−8.9** ± 8.3^*^	**0.1** ± **4**	**-**	**1.1** ± **18.6**	**−8.5** ± 14.5^*^	**−0.9** ± **0.6**	**-**	**−3.3** ± **23**	**-0.4**	**1**	**-**	**4.4**
Baseline	37.2 ± 17.2	NR	24.7 ± 10.1	NR	41.9 ± 21.1	NR	19.9 ± 10.1	NR				
Last visit	18.9 ± 12.8	NR	24.3 ± 10.4	NR	26.6 ± 16.7	NR	22.5 ± 11.5	NR				
**Difference**^**a**^	**−18.3** ± 17.8^*^	**-**	**−0.4** ± **8.3**	**-**	**−15.3** ± 15.3^*^	**-**	**2.6** ± **12.3**	**-**	−3^*^	**-**	**−3**	**-**
Baseline	37.2 ± 17.2	NR	24.7 ± 10.1	NR	38.8 ± 17.1	NR	20.4 ± 10.5	NR				
Last visit	18.9 ± 12.8	NR	24.3 ± 10.4	NR	39.6 ± 21.2	NR	18.6 ± 11.5	NR				
**Difference**^**a**^	**−18.3** ± 17.8^*^	**-**	**−0.4** ± **8.3**	**-**	**0.8** ± **15.2**	**-**	**−1.8** ± **9.6**	**-**	−17.5^*^	**-**	**1.4**	**-**
Baseline	39.8 ± 4.9	4.1 ± 0.7	NR	NR	40.1 ± 5.6	4.1 ± 0.6	NR	NR				
Last visit	32.9 ± 4.9	5.9 ± 0.8	NR	NR	22.5 ± 3.1	7.3 ± 0.9	NR	NR				
**Difference**^**a**^	**−6.9** ± 4.9^*^	**1.8** ± 0.8^*^	**-**	**-**	**−17.6** ± 4.4^*^	**3.2** ± 0.8^*^	**-**	**-**	10.7^*^	−1.4^*^	**-**	**-**

*CLDE* contact lens-related dry eye, *CMC* carboxymethyl cellulose, *IPL* intense pulse light, *TBUT* tear film break-up time, *LLT* lipid layer thickness, *NR* not reported, OSDI ocular surface disease index, ST Schirmer test.

^a^ Defined as “Last visit – Baseline.”

^b^ Defined as “Eyelid warming therapies _(Last visit – Baseline)_ – Controls _(Last visit – Baseline).”_

^c^ Lipiflow^®^ (Single 12-min session) Vs. No intervention.

^d^ Bruder Moist Heat Compress^®^ (once daily for 10 min) Vs. Hot towel (twice daily for 10 min).

^e^ Bruder Moist Heat Compress^®^ (twice daily for 10 min) Vs. Hot towel (twice daily for 10 min).

^f^ Steam Eye Mask^®^ (once daily for 30 min) Vs. Refresh Optive^®^ (0.5% CMC, single-use vial once daily).

^g^ Steam Eye Mask^®^ (once daily for 30 min) Vs. No intervention.

^h^ Steam Eye Mask^®^ (once daily for 15 min) Vs. IPL Stellar M22^®^ (two sessions spaced 3 weeks apart).

^i^ All studies performed TBUT with fluorescein, except Xu et al. [35], who employed non-invasive TBUT.

^*^ Statistical significance with a *P* value < 0.05.

Regarding intra-group outcome measures, Blackie et al. [13] reported a single 12-min session of LipiFlow® decreased OSDI scores (−26.2 ± 20.3 points; *p* not reported) and improved FTBUT (1.7 ± 3.4 s; *p* not reported). In contrast, the non-intervention group showed only slight reductions in OSDI scores (−3.3 ± 22.6 points; *p* not reported), whereas FTBUT remained unchanged. Tichenor et al. [14] (A) reported that using the Bruder Moist Heat Compress® once daily for 10 min decreased the OSDI score (−16.3 ± 17.7 points; *p* < 0.05) and led to improvements in LLT (1.4 ± 21.7 nm; *p* not reported). Tichenor et a.l [14] (B) also found that applying the Bruder Moist Heat Compress® twice daily for 10 min resulted in a significant reduction in OSDI score (−8.9 ± 8.3 points; *p* < 0.05), along with a similar increase in LLT (1.1 ± 18.6 nm; *p* not reported). Conversely, the hot towel group showed smaller reductions in OSDI scores (−8.5 ± 14.5 points; *p* < 0.05), accompanied by a worsening of LLT (−3.3 ± 23 points; *p* not reported). Additionally, FTBUT remained similar across all groups. Jeon and Park [34] (A and B) observed that using the Steam Eye Mask® once daily for 30 min resulted in reductions in OSDI scores (−18.3 ± 17.8 points; *p* < 0.001), while the Schirmer test findings remained unchanged. Similar results were reported in OSDI scores (−15.3 ± 15.3 points; *p* < 0.001) after the instillation of Refresh Optive®, whereas Schirmer test results improved (2.6 ± 12.3 mm; *p* = 0.343) [34]. However, OSDI scores remained unchanged in the non-intervention group, while Schirmer test worsened slightly (−1.8 ± 9.6 mm; *p* < 0.05). Xu et al. [35] found that applying the Steam Eye Mask® once daily for 15 min decreased OSDI scores (−6.9 ± 4.9 points; *p* < 0.001) and led to improvements in NIBUT (1.8 ± 0.8 s; *p* < 0.001). In contrast, the IPL group showed large reductions in OSDI scores (−17.6 ± 4.4 points; *p* < 0.001), along with a more pronounced increase in NIBUT (3.2 ± 0.8 s; *p* < 0.001).

Regarding inter-group outcome measures, studies reported favourable trends in alleviating CLDE with eyelid warming therapies, although most of these improvements did not reach statistical significance. Tichenor et al. [14] (A) reported a reduction of −7.8 points in OSDI scores (*p* > 0.05) accompanied by an increase of 0.7 s in FTBUT (*p* > 0.05) and 4.7 nm in LLT (*p* > 0.05). However, Tichenor et al. [14] (B) showed a reduction of only −0.4 points in OSDI scores (*p* > 0.05), with increases of 1 s in FTBUT (*p* > 0.05) and 4.4 nm in LLT (*p* > 0.05). Jeon and Park [34] (A) reported a reduction of −3 points in the OSDI scores (*p* < 0.001). In contrast, both Jeon and Park [34]. (B) and Blackie et al. [13] observed larger reductions in OSDI scores, with decreases of −17.5 points (*p* < 0.001) and −22.9 points (*p* < 0.001), respectively. In addition, Blackie et al. [13] also reported the largest improvement in FTBUT, with a value of 2 s (*p* < 0.001). However, contrasting results were observed for the Schirmer test between the studies. Xu et al. [35] was the only investigation that showed favourable outcomes for the control group, with a reduction of −10.7 points in the OSDI score (*p* < 0.001), accompanied by an increase of 1.4 s in NIBUT (*p* < 0.001).

#### Comfortable contact lens wearing time

Intra- and inter-group efficacy of eyelid warming therapies on CL wearing time are presented in Table 3. Regarding intra-group outcome measures, Blackie et al. [13] reported that a single 12-min session of LipiFlow® increased CL wearing time (3.7 ± 3.2 h; *p* not reported), while it remained unchanged in the non-intervention group. Tichenor et al. [14] (A and B) reported that using the Bruder Moist Heat Compress® once (2.2 ± 3.2 h; *p* not reported) or twice daily (1.4 ± 1.5 h; *p* not reported) for 10 min increased CL wearing time. However, the hot towel group showed similar improvements in CL wearing time (1.7 ± 2.5 points; *p* > 0.05). Regarding inter-group outcome measures, Blackie et al. [13] reported favourable results for LipiFlow®, showing an average increase of 4 h in CL wearing time compared with the non-intervention group (*p* < 0.001). However, contrasting results were observed for comfortable CL wear time between Tichenor et al. [14] (A and B).

**Table 3:** Intra-group and inter-group differences in comfortable CL wear time of eyelid warming therapies Vs. controls.

Author (Date)		Comfortable CL wear time, hours		Inter-group differences^e^
		Eyelid warming therapies^e^	Control	
Blackie et al. [13] 2018^a^	Baseline	NR	NR	
	Last visit	NR	NR	
	**Difference**^**d**^	**3.7** ± **3.2**	**−0.3** ± **2.2**	4^*^
Tichenor et al. [14] (A) 2019^b^	Baseline	8.0 ± 3.9	5.9 ± 3.2	
	Last visit	10.2 ± 2.4	7.6 ± 1.6	
	**Difference**^**d**^	**2.2** ± **3.2**	**1.7** ± **2.5**	**0.5**
Tichenor et al. [14] (B) 2019^c^	Baseline	7.8 ± 3.0	5.9 ± 3.2	
	Last visit	9.2 ± 2.8	7.6 ± 1.6	
	**Difference**^**d**^	**1.4** ± **1.5**	**1.7** ± **2.5**	**−0.3**

*CL* contact lens, *NR* not reported.

^*^ Statistical significance with a *P*-value < 0.05.

^a^ Lipiflow^®^ (Single 12-min session) Vs. No intervention.

^b^ Bruder Moist Heat Compress^®^ (once daily for 10 min) Vs. Hot towel (twice daily for 10 min).

^c^ Bruder Moist Heat Compress^®^ (twice daily for 10 min) Vs. Hot towel (twice daily for 10 min).

^d^ Defined as “Last visit – Baseline.”

^e^ Defined as “Eyelid warming therapies _(Last visit – Baseline)_ – Controls _(Last visit – Baseline).”_

### Safety of Eyelid Warming Therapies

In terms of the safety of eyelid warming therapies, Blackie et al. [13] observed slit-lamp findings after a single 12-min session of LipiFlow®, including eyelid and conjunctival oedema, conjunctival hyperaemia and superficial punctate keratitis. Tichenor et al. [14] (A and B) observed no significant changes in best-corrected visual acuity (BCVA) following use of the Bruder Moist Heat Compress® once or twice daily for 10 min. Furthermore, no adverse events were documented during the study. Similarly, Xu et al. [35] reported the absence of adverse events, as well as no significant changes in BCVA and intraocular pressure after the use of the Steam Eye Mask® once daily for 15 min. In contrast, Jeon and Park [34] did not evaluate the safety of the Steam Eye Mask®.

### Meta-Analyses, Subgroup Analysis and Sensitivity Analyses

#### Contact lens-related dry eye

##### Ocular surface disease index

**Fig. 3:**
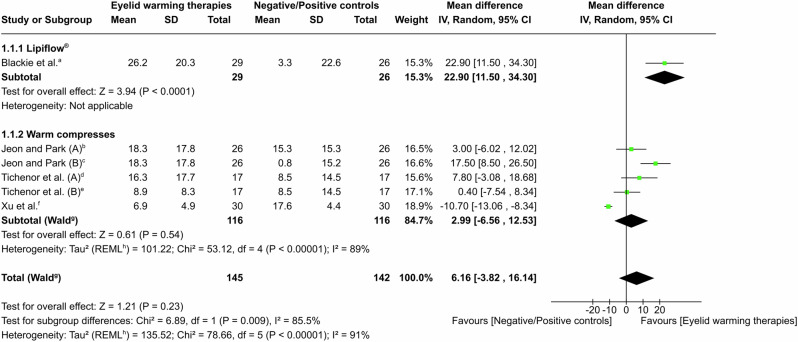
Meta-analysis. Overall efficacy of eyelid warming therapies (1.1.1 LipiFlow®and 1.1.2 Warm compresses) compared to negative/positive controls for ocular surface disease index (OSDI) measurements and Forest plot showing the mean difference (MD), 95% confidence intervals (CI) and p-value for the change in OSDI scores. A random effects model was performed, revealing no significant differences between both groups. However, LipiFlow®achieved a significant decrease in OSDI scores compared to warm compresses in the subgroup analysis. ^a^Lipiflow®(Single 12-min session) Vs. No intervention. ^b^Steam Eye Mask®(once daily for 30 min) Vs. Refresh Optive®(0.5% CMC, single-use vial once daily). ^c^Steam Eye Mask®(once daily for 30 min) Vs. No intervention. ^d^Bruder Moist Heat Compress®(once daily for 10 min) Vs. Hot towel (twice daily for 10 min). ^e^Bruder Moist Heat Compress®(twice daily for 10 min) Vs. Hot towel (twice daily for 10 min). ^f^Steam Eye Mask®(once daily for 15 min) Vs. IPL Stellar M22®(two sessions spaced 3 weeks apart). ^g^CI calculated by Wald-type method. ^h^Tau^2^calculated by the Restricted Maximum-Likelihood method.

In the overall analysis, eyelid warming therapies did not lead to a significant reduction in OSDI scores compared with negative/positive controls (MD: 6.16; 95% CI: −3.82 to 16.14; *I*^2^ = 91%; *p* = 0.23) (Fig. 3). However, LipiFlow® (MD: 22.90; 95% CI: 11.50 to 34.40; *I*² not applicable; *p* < 0.001) achieved a significant decrease in OSDI scores compared to warm compresses (MD: 2.99; 95% CI: -6.56 to 12.53; *I*² = 89%; p = 0.54), as shown in the subgroup analysis (*p* = 0.009) (Fig. 3).

**Fig. 4:**
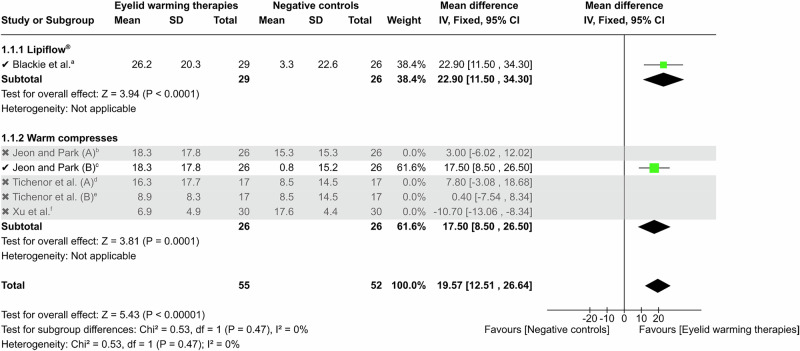
Sensitivity analysis. Efficacy of eyelid warming therapies (1.1.1 LipiFlow® and 1.1.2 Warm compresses) compared to negative controls for the Ocular Surface Disease Index (OSDI). Forest plot showing the mean difference (MD), 95% confidence intervals (CI) and p-value for the change in OSDI scores. A fixed effects model was performed, revealing significant differences between both groups. However, no significant differences in the reduction of OSDI scores between LipiFlow® and warm compresses was identified in the subgroup analysis. ^a^Lipiflow® (Single 12-min session) Vs. No intervention. ^c^Steam Eye Mask® (once daily for 30 min) Vs. No intervention.

**Fig. 5:**
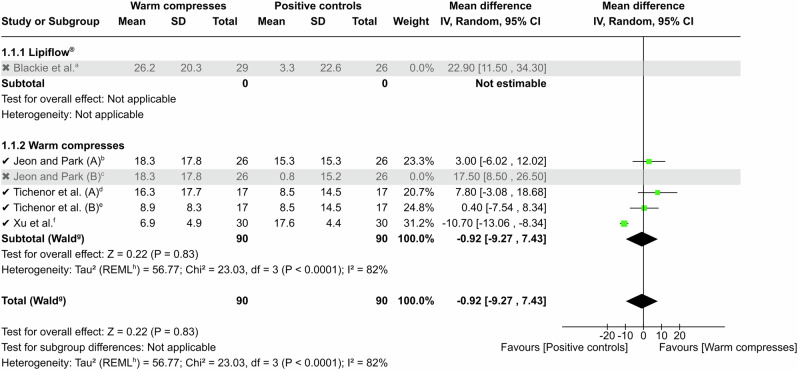
Sensitivity analysis. Efficacy of eyelid warming therapies (1.1.2 warm compresses) compared to positive controls for the Ocular Surface Disease Index (OSDI). Forest plot showing the mean difference (MD), 95% confidence intervals (CI) and p-value for the change in OSDI scores. A random effects model was performed, revealing no significant differences between both groups. ^b^Steam Eye Mask® (once daily for 30 min) Vs. Refresh Optive® (0.5% CMC, single-use vial once daily). ^d^Bruder Moist Heat Compress® (once daily for 10 min) Vs. Hot towel (twice daily for 10 min). ^e^Bruder Moist Heat Compress® (twice daily for 10 min) Vs. Hot towel (twice daily for 10 min). ^f^Steam Eye Mask® (once daily for 15 min) Vs. IPL Stellar M22® (two sessions spaced 3 weeks apart). ^g^CI calculated by Wald-type method. ^h^Tau^2^calculated by the Restricted Maximum-Likelihood method.

**Fig. 6:**
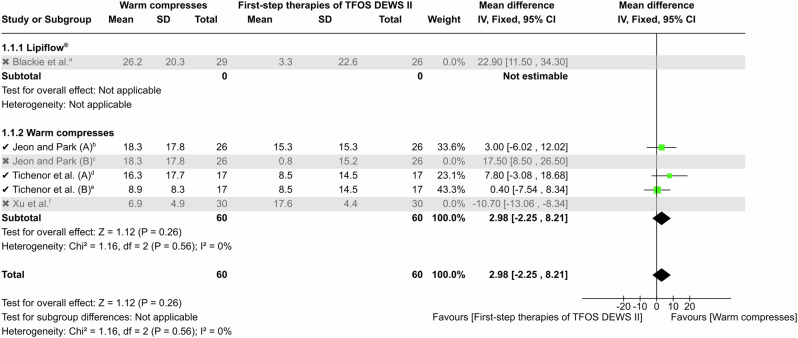
Sensitivity analysis. Efficacy of eyelid warming therapies (1.1.2 Warm compresses) compared to first-step therapies recommended by the Tear Film and Ocular Surface Dry Eye Workshop II (TFOS DEWS II) for ocular surface disease index (OSDI). Forest plot showing the mean difference (MD), 95% confidence intervals (CI) and *p*-value for the change in OSDI scores. A fixed effects model was performed, revealing significant differences between both groups. ^b^Steam Eye Mask®(once daily for 30 min) Vs. Refresh Optive®(0.5% CMC, single-use vial once daily). ^d^Bruder Moist Heat Compress®(once daily for 10 min) Vs. Hot towel (twice daily for 10 min). ^e^Bruder Moist Heat Compress®(twice daily for 10 min) Vs. Hot towel (twice daily for 10 min).

In contrast, sensitivity analysis showed that eyelid warming therapies led to a significant reduction in OSDI scores when compared to negative controls (MD: 19.57; 95% CI: 12.51 to 26.64; *I*^2^ = 0%; *p* < 0.001) (Fig. 4). Furthermore, no significant differences in the reduction of OSDI scores were observed between Lipiflow® (MD: 22.90; 95% CI: 11.50 to 34.40; *I*^2^ not applicable; *p* < 0.001) and warm compresses (MD: 17.50; 95% CI: 8.50 to 26.50; *I*^2^ not applicable; *p* = 0.001) in the subgroup analysis (*p* = 0.53) (Fig. 4). Warm compresses did not produce a significant decrease in OSDI scores when compared to positive controls (MD: −0.92; 95% CI: −9.27 to 7.43; *I*^2^ = 82%; *p* = 0.83) (Fig. 5) or first-step therapies recommended by the Tear Film and Ocular Surface Dry eye Workshop II (TFOS DEWS II) (MD: 2.98; 95% CI: −2.25 to 8.21; *I*^2^ = 0%; *p* = 0.26) (Fig. 6).

##### Tear film break-up time

**Fig. 7:**
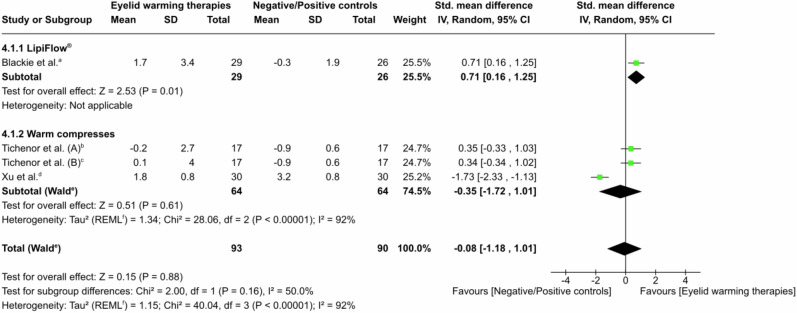
Meta-analysis. Overall efficacy of eyelid warming therapies (1.1.1 LipiFlow® and 1.1.2 Warm compresses) compared to negative/positive controls for tear film break-up time (TBUT). Forest plot showing the standardised mean difference (SMD), 95% confidence intervals (CI) and p-value for the change in TBUT. A random effects model was performed, revealing no significant differences between both groups. In addition, no significant differences in the increase of TBUT between LipiFlow® and warm compresses were observed in the subgroup analysis. ^a^Lipiflow® (Single 12-min session) Vs. No intervention. ^b^Bruder Moist Heat Compress® (once daily for 10 min) Vs. Hot towel (twice daily for 10 min). ^c^Bruder Moist Heat Compress® (twice daily for 10 min) Vs. Hot towel (twice daily for 10 min). ^d^Steam Eye Mask® (once daily for 15 min) Vs. IPL Stellar M22® (two sessions spaced 3 weeks apart). ^e^CI calculated by Wald-type method. ^f^Tau^2^calculated by the Restricted Maximum-Likelihood method.

**Fig. 8:**
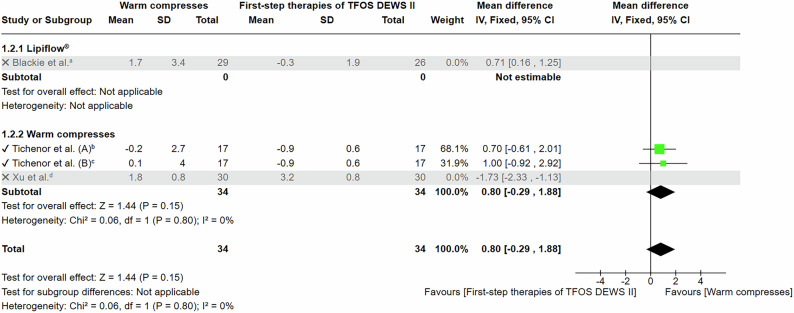
Sensitivity analysis. Efficacy of eyelid warming therapies (1.1.2 Warm compresses) to first-step therapies recommended by the Tear Film and Ocular Surface Dry Eye Workshop II (TFOS DEWS II) for tear film break-up time (TBUT). Forest plot showing the mean difference (MD), 95% confidence intervals (CI) and p-value for the change in TBUT. A fixed effects model was performed, revealing no significant differences between both groups. ^b^Bruder Moist Heat Compress®(once daily for 10 min) Vs. Hot towel (twice daily for 10 min). ^c^Bruder Moist Heat Compress®(twice daily for 10 min) Vs. Hot towel (twice daily for 10 min).

In the overall analysis, eyelid warming therapies did not lead to a significant increase in TBUT compared to negative/positive controls (SMD: −0.08; 95% CI: −1.18 to 1.01; *I*^2^ = 92%; *p* = 0.88) (Fig. 7). In addition, no significant differences in the increase of TBUT were observed between LipiFlow® (SMD: 0.71; 95% CI: 0.16–1.25; *I*^2^ not applicable; *p* = 0.01) and warm compresses (SMD: −0.35; 95% CI: −1.72 to 1.01; *I*^2^ = 92%; *p* = 0.61) in the subgroup analysis (*p* = 0.16) (Fig. 7). Warm compresses also did not produce a significant increase in TBUT when compared to first-step therapies recommended by TFOS DEWS II (MD: 0.80; 95% CI: −0.29 to 1.88; *I*^2^ = 0%; *p* = 0.15) (Fig. 8).

##### Schirmer test

**Fig. 9:**
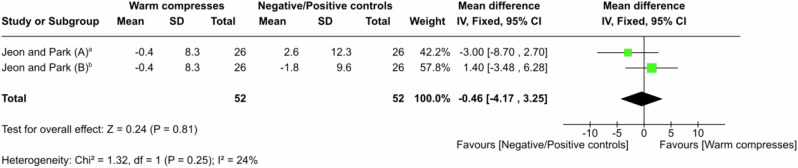
Meta-analysis. Efficacy of warm compresses compared to negative/positive controls for the Schirmer test. Forest plot showing the mean difference (MD), 95% confidence intervals (CI) and *p*-value for the change in Schirmer test. A fixed effects model was performed, revealing no significant differences between both groups. ^a^Steam Eye Mask® (once daily for 30 min) Vs. Refresh Optive® (0.5% CMC, single-use vial once daily). ^b^Steam Eye Mask® (once daily for 30 min) Vs. No intervention.

Warm compresses did not produce a significant increase in Schirmer test findings compared with the negative/positive controls (MD: −0.46; 95% CI: −4.17 to 3.25; *I*^2^ = 24%; *p* = 0.81) (Fig. 9).

##### Lipid layer thickness

**Fig. 10:**
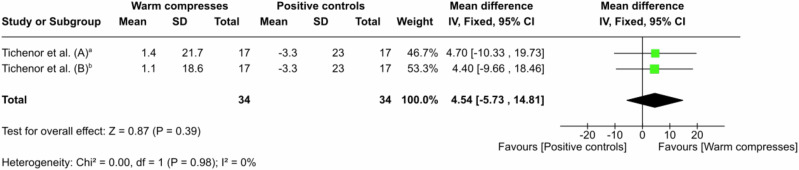
Meta-analysis. Efficacy of warm compresses compared to positive controls for lipid layer thickness (LLT). Forest plot showing the mean difference (MD), 95% confidence intervals (CI) and *p*-value for the change in LLT. A fixed effects model was performed, revealing no significant differences between both groups. ^a^Bruder Moist Heat Compress®(once daily for 10 min) Vs. Hot towel (twice daily for 10 min). ^b^Bruder Moist Heat Compress®(twice daily for 10 min) Vs. Hot towel (twice daily for 10 min).

Warm compresses did not achieve a significant increase in LLT compared to positive controls (MD: 4.54; 95% CI: −5.73 to 14.81; *I*^2^ = 0%; *p* = 0.39) (Fig. 10).

##### Comfortable contact lens wearing time

**Fig. 11:**
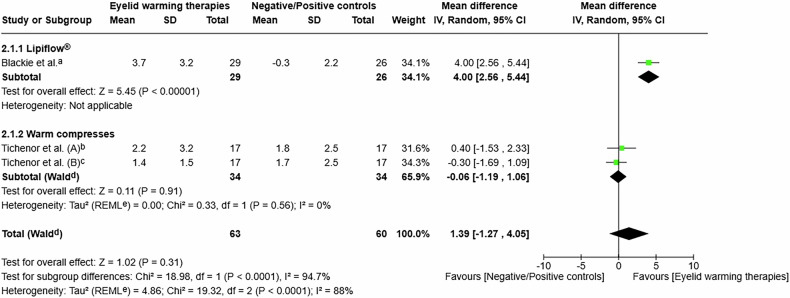
Meta-analysis. Overall efficacy of eyelid warming therapies (1.1.1 LipiFlow® and 1.1.2 Warm compresses) compared to negative/positive controls for comfortable contact lens (CL) wearing time. Forest plot showing the mean difference (MD), 95% confidence intervals (CI) and *p*-value for the change in comfortable CL wearing time. A random effects model was performed, revealing no significant differences between both groups. However, LipiFlow® achieved a significant increase of comfortable CL wearing time compared to warm compresses in the subgroup analysis. ^a^Lipiflow® (Single 12-min session) Vs. No intervention. ^b^Bruder Moist Heat Compress® (once daily for 10 min) Vs. Hot towel (twice daily for 10 min). ^c^Bruder Moist Heat Compress® (twice daily for 10 min) Vs. Hot towel (twice daily for 10 min). ^d^CI calculated by the Wald-type method. ^e^Tau^2^calculated by Restricted Maximum-Likelihood method.

In the overall analysis, eyelid warming therapies did not lead to a significant increase in comfortable CL wearing time compared to negative/positive controls (MD: 1.39; 95% CI: −1.27 to 4.05; *I*^2^ = 88%; *p* = 0.31) (Fig. 11). However, LipiFlow® (MD: 4.00; 95% CI: 2.56 to 5.44; I *I*^2^ not applicable; *p* < 0.001) achieved a significant increase in comfortable CL wearing time compared to warm compresses (MD: −0.06; 95% CI: −1.19 to 1.06; *I*^2^ = 0%; *p* = 0.91), as shown in the subgroup analysis (*p* < 0.0001) (Fig. 11).

##### Risk of bias

**Fig. 12:**
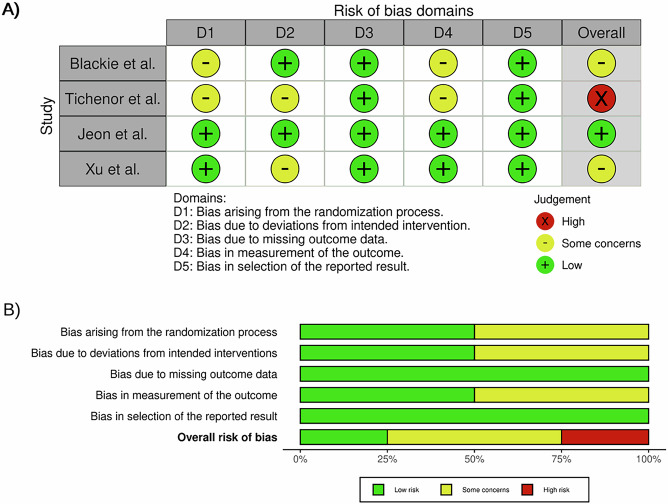
Risk of bias assessment: **A** Risk of bias summary of the included studies [13, 14, 34, 35] with traffic light plot. The traffic lights represent the author’s risk of bias judgement in each domain (D) used to assess the quality of the studies. **B** Overall risk of bias summary of the domains with bar plot. Bars represent the overall author’s risk of bias judgement in each domain presented as percentages.

The risk of bias summary for the RCTs is presented in Fig. 12A. The risk of bias assessment was categorised into three evidence levels: (1) those with a low risk of bias (Jeon and Park [34]), (2) those with some concerns (Blackie et al. [13] and Xu et al. [35]) and (3) those with a high risk of bias (Tichenor et al. [14]). The overall summary of the risk of bias across the domains assessed in each study is shown in Fig. 12B. The items used to evaluate the risk of bias indicated that the overall risk of bias was classified as low in 25% of the cases, unclear in 50% and high in 25%.

## Discussion

CLDE, frequently associated with MGD, remains a significant challenge despite advancements in CL materials and design [7–9]. Although eyelid warming therapies have been shown to be effective and safe in managing MGD-related dry eye [18–21], their specific impact on CLDE and comfortable CL wearing time does not appear to have been investigated thoroughly. Therefore, the aim of this study was to analyse the efficacy and safety of eyelid warming therapies in CLDE and in relation to comfortable CL wearing time.

These findings suggest that eyelid warming therapies do not provide superior efficacy in reducing OSDI scores, increasing TBUT or extending comfortable CL wearing time when compared with the control group, which comprised both negative (no intervention) and positive controls (hot towels, artificial tears or IPL). Among eyelid warming therapies, LipiFlow® produced a significant reduction in OSDI scores, along with a significant increase in comfortable CL wearing time compared with warm compresses, whereas no significant differences were observed between these two therapies regarding TBUT. Nonetheless, it is important to detail the methodological differences in the included studies. Blackie et al. [13]. compared LipiFlow® with a negative control (no intervention), whereas the remaining studies [14, 34, 35] used warm compresses, comparing them with both negative (no intervention) and positive controls (hot towels, artificial tears or IPL). In addition, the characteristics of the sample was also a potential factor that may have contributed to these differences. For instance, the prevalence of MGD ranges from 3.5% to 70%, increases with age and is more prevalent in the Asian population [36, 37]. Among the included studies, 56.5% of the sample was Asian, while the average age was 26.6 ± 10.8 years. In addition, it is important to note that only Blackie et al. [13]. and Xu et al. [35]. included MGD diagnosis as part of their inclusion criteria. However, although participants in the other studies included in this systematic review exhibited symptoms and signs of dry eye, it remains unclear whether they were diagnosed with MGD. Given that eyelid warming therapies have been shown to be particularly effective in the presence of MGD [18–21], it is likely that CLDE and reduced CL wearing time were more influenced by factors related to the CLs, such as replacement schedule, care system, material and design. Therefore, these methodological and sample-related differences may influence assessment of the overall efficacy of eyelid warming therapies, as well as the comparison between LipiFlow® and warm compresses, potentially influencing the observed effect sizes. For this reason, a sensitivity analysis was performed to evaluate the efficacy of eyelid warming therapies separately, in comparison with both negative and positive controls. However, it could only be conducted for the comparison between eyelid warming therapies and negative controls with respect to the OSDI score. This analysis suggests that eyelid warming therapies result in a significantly greater reduction in OSDI scores than negative controls (no intervention). Furthermore, the magnitude of these reductions was similar for both LipiFlow® and warm compresses. Regarding the latter therapy, meta-analyses showed that they did not produce significant reductions in OSDI scores when compared with the control group, which included both negative (no intervention) and positive controls (hot towels, artificial tears or IPL). Similarly, warm compresses also did not produce significant improvements in TBUT compared with positive controls (hot towels and IPL), or Schirmer test findings, when compared with negative (no intervention) and positive controls (hot towels), or for LLT and comfortable CL wearing time compared with a positive control (hot towels). However, the results reported for OSDI and TBUT may have been biased due to the inclusion of IPL as a positive control. Indeed, a recent RCT revealed that IPL is effective in CL wearers with MGD-related dry eye, achieving significant reductions in OSDI scores and an increased NIBUT [38]. Furthermore, other RCTs have evaluated the efficacy of IPL compared with warm compresses. Yan et al. [39] reported that IPL combined with meibomian gland expression resulted in a more pronounced reduction in Standard Patient Evaluation of Eye Dryness (SPEED) scores, along with superior improvements in TBUT and meibomian gland yielding secretion score, compared with warm compresses followed by meibomian gland expression. Similarly, a further study [40] found that IPL achieved superior results than warm compresses, followed by eyelid massage in tear secretion function, tear meniscus height and meibomian gland quality score. Therefore, a sensitivity analysis was conducted, excluding the findings of Xu et al. [35], to assess specifically the efficacy of warm compresses in comparison with hot towels and artificial tears—therapies recommended as a first-step in the management of DED by the TFOS DEWS II. Although these analyses led to an increased effect size (OSDI: from 0.22 to 1.12 and TBUT: from 0.15 to 1.44) and a reduction in heterogeneity (OSDI: from 82% to 0% and TBUT: from 92% to 0%), warm compresses did not produce significant differences in OSDI or TBUT when compared with hot towels and artificial tears.

Although several systematic reviews with meta-analyses have evaluated the effects of LipiFlow® compared to warm compresses [41–43], as well as warm compresses compared to hot towels [44], the included studies did not specifically involve CL wearers with MGD-related dry eye. Therefore, the results of these reviews on OSDI and TBUT are not directly comparable with the findings presented here. CL wearers tend to present with more severe MGD than non-CL wearers due to chronic mechanical trauma to the meibomian glands caused by increased friction between the CL and the palpebral conjunctiva [45]. In contrast, to the best of our knowledge, no systematic reviews with meta-analyses have evaluated the effects of warm compresses compared with artificial tears. A recent RCT by Arroyo-Del-Arroyo et al. [46] assessed the sequential implementation of CLDE management strategies. This study suggested that warm compresses combined with eyelid massage and cleansing significantly reduce CLDE, whereas the simultaneous administration of artificial tears did not provide additional benefit [46]. This is consistent with Caffery et al. [47], who reported that very low-certainty evidence supports the proposal that artificial tears may improve CLDE.

Regarding eyelid warming therapies’ safety, most studies reported no adverse events, as well as no significant changes in either BCVA or intraocular pressure. In addition, all therapies demonstrated satisfactory tolerability profiles. Blackie et al. [13] was the only study to report slit-lamp findings following a single 12-min session of LipiFlow®. However, these included transient changes that resolved without requiring medical intervention and were consistent with observations from previous studies [48, 49]. It is important to note that most studies did not provide detailed data on safety parameters, including adverse events, BCVA, intraocular pressure, corneal topography, slit-lamp biomicroscopy or dilated funduscopy. As a result, a meta-analysis could not be performed, which limits the comprehensive evaluation of the safety profile of eyelid warming therapies in CL wearers with MGD-related dry eye.

### Strengths and Limitations

To the best of our knowledge, this is the first systematic review with meta-analysis that evaluated the efficacy and safety of eyelid warming therapies in CLDE, as well as assessed comfortable CL wearing time. However, several limitations should be acknowledged. One major limitation is the small number of included studies, with only four RCTs. Furthermore, the diagnostic criteria for CLDE varied across the studies, and none explicitly indicated adherence to the recommendations of the Subcommittee on Epidemiology of the TFOS International Workshop on CL discomfort [4]. Further, some did not include MGD-related dry eye in their inclusion criteria. This heterogeneity in study populations, combined with variations in both treatment and control intervention protocols across the studies, contributed to the substantial heterogeneity observed in the meta-analyses (*I*² = 59% ± 39.1%), which persisted at a moderate level despite conducting a sensitivity analysis (*I*^2^ = 20.5 ± 35.5%). Therefore, these limitations may impact the strength and generalisability of the conclusions that can be drawn.

Another limitation is that two of the four RCTs declared conflicts of interest and were industry-funded. In addition, most of the RCTs were assessed as having a high or unclear risk of bias. According to the Cochrane Handbook for Systematic Reviews of Interventions, RCTs with a higher risk of bias are more likely to overestimate treatment effects [50]. Moreover, this effect may be exacerbated when authors have conflicts of interest with the industry distributing the product under investigation, or when the study is industry-funded [51]. A recent Cochrane methodological review conducted by Lundh et al. [52] analysed 75 RCTs, examining the association between industry funding and study outcomes, concluding that industry-funded RCTs were more likely to report statistically significant results and positive conclusions. Therefore, the results reported by the RCTs included in the present meta-analysis may have overestimated the effect size, which emphasises the need for caution when interpreting the efficacy of eyelid warming therapies in CLDE and comfortable CL wearing time. Nevertheless, although the scientific literature also suggests that industry-funded systematic reviews tend to have lower methodological quality than those without industry funding [53], the authors of this systematic review have no conflicts of interest and the study was not industry-funded. Furthermore, it was conducted in strict adherence to the PRISMA guidelines [26]. Thus, despite the limitations mentioned, the review process was conducted with methodological rigour and transparency at all stages.

Although this study synthesises all currently available scientific literature on the efficacy and safety of eyelid warming therapies for CLDE and comfortable CL wearing time, novel designs such as iLux® and TearCare® were not included due to the lack of RCTs. iLux® is a hand-held device that simultaneously delivers controlled heat and mechanical pressure to the meibomian glands, enabling liquefaction and expression of their secretions. In contrast, TearCare® employs externally adhered thermal patches on the upper and lower eyelids, allowing the patient to blink naturally during treatment. Following the controlled heating phase, the eye care practitioner manually expresses the meibomian glands using meibomian gland expressor forceps.

Moreover, the limited available scientific literature precluded several analyses that could have provided relevant information. Specifically, it was not possible to assess the efficacy of eyelid warming therapies compared with positive controls for OSDI and TBUT, nor their efficacy compared with negative controls for TBUT and comfortable CL wearing time. In addition, the efficacy of warm compresses could not be assessed using the Schirmer test in comparison with either negative or positive controls. Regarding the analysed variables, all of the studies included used the OSDI questionnaire to assess CL discomfort, whereas only Blackie et al. [13] also employed the Contact Lens Dry Eye Questionnaire (CLDEQ). This tendency toward a greater use of the OSDI in studies involving CL wearers was reported previously by Jalbert et al. [54]. These authors reviewed the use of different questionnaires for evaluating CL discomfort between 2009 and 2015, reporting that the OSDI questionnaire was used in 13 studies, while the CLDEQ was used in seven [54]. Although the OSDI questionnaire is widely used for assessing DED [55], it does not specifically assess the relationship between dry eye symptoms and CL use [54, 56]. Therefore, its extensive use in studies including CL wearers remains uncertain, as the scientific literature does not appear to have evaluated its sensitivity and specificity for CL discomfort. In contrast, the CLDEQ represents the best-validated questionnaire for assessing both the frequency and intensity of CL discomfort [54, 57]. In addition, the CLDEQ has demonstrated a predictive efficiency of 1.44, with a sensitivity of 83% and specificity of 67% [58]. Therefore, the use of the OSDI questionnaire by the RCTs included in the present study may not reflect CL discomfort as accurately as CLDEQ, potentially introducing measurement bias that could influence the results of the meta-analysis. Furthermore, the meta-analyses also did not include variables related to meibomian gland function, as the RCTs primarily assessed MGD through different parameters, such as meibomian gland yielding secretion score, meibomian gland quality, meibomian gland expression and the number of functional meibomian glands.

Overall, given these limitations, further RCTs not funded by the industry and strictly adhering to the Consolidated Standards of Reporting Trials (CONSORT) guidelines are needed to evaluate the efficacy and safety of eyelid warming therapies rigorously in CLDE and comfortable CL wearing time [59]. These studies should not only evaluate warm compresses and LipiFlow®, but expand to include novel eyelid warming devices such as iLux® and TearCare®. Particular emphasis should be placed on the use of standardised diagnostic criteria for characterising CL wearers with MGD-related dry eye, in accordance with the recommendations from the TFOS International Workshops on CL discomfort [4] and MGD [60]. Furthermore, to assess both the therapeutic effects and safety profile of these therapies in CL wearers with MGD-related dry eye better, future studies should include validated symptom questionnaires such as the CLDEQ [57]; meibum-related continuous variables, including meibomian gland yielding secretion score, meibomian gland yielding liquid secretion and meibomian gland yielding clear secretion [61]. Safety parameters such as ocular and non-ocular treatment-emergent adverse events and treatment-related AEs should also be included [62].

## Conclusion

Based on the data included in the meta- and sensitivity analyses, the efficacy of eyelid warming therapies for alleviating CLDE and improving comfortable CL wearing time is limited. These therapies produce a significant reduction in OSDI scores compared to no intervention, with similar results being observed between the LipiFlow® and warm compresses. However, this result should be interpreted with caution due to the heterogeneity among the included studies, potential risk of bias, conflicts of interest and industry funding. Furthermore, the lack of studies specifically addressing this topic prevented relevant comparisons between eyelid warming therapies and both negative (no intervention) and positive controls (hot towels, artificial tears or IPL) across various clinical parameters. Similarly, a comprehensive evaluation of the safety profile of these therapies is hindered by the absence of standardised safety reporting among the included studies. Therefore, current evidence is insufficient to support the effectiveness of eyelid warming therapies in improving OSDI, TBUT, Schirmer test results, LLT or comfortable CL wearing time in CL wearers with MGD-related dry eye, as well as to establish their safety adequately. Further well-designed RCTs adhering to CONSORT guidelines are needed to determine the efficacy and safety of eyelid warming therapies in CL wearers with MGD-related dry eye.

## Data Availability

The data sets analysed during the current study are available from the corresponding author upon reasonable request.
